# Supplementary material for: Empirical methods for controlling false positives and estimating confidence in ChIP-Seq peaks
Source: BMC Bioinformatics. 2008 Dec 5;9:523. doi: 10.1186/1471-2105-9-523 (PMC2628906; doi:10.1186/1471-2105-9-523)
Supplement: Additional File 1 — A variety of html documents from the USeq web site detailing the available applications, their best usage, output file type descriptions, command line menus, etc. [file 1471-2105-9-523-S1.zip › USeqUserGuides/JavaDoc.zip!/JavaDoc/meme/class-use/MotifHit.html]

Uses of Class meme.MotifHit


|  |  |  |
| --- | --- | --- |
| **Overview** \| **Package** \| **Class** \| **Use** \| **Tree** \| **Deprecated** \| **Index** \| **Help** | |  |
| PREV   NEXT | **FRAMES**    **NO FRAMES**     **All Classes** |  |


---


## **Uses of Class meme.MotifHit**

| Packages that use MotifHit | |
| --- | --- |
| **meme** | Wrapper for meme tweaked for processing multiple rounds of SELEX oligos. |

Uses of MotifHit in meme

| Methods in meme that return MotifHit | |
| --- | --- |
| `MotifHit[]` | `MotifScanner.scoreSequence(double cutOff, java.lang.String seq)`             Returns an array of MotifHits that are > or = to the loglikelihood cutOff. |

| Methods in meme with parameters of type MotifHit | |
| --- | --- |
| `static int` | `MotifScanner.calculateMaxCluster(MotifHit[] hits, int window)`             Calculates the maximum number of hits that occur within a given window size. |

---


|  |  |  |
| --- | --- | --- |
| **Overview** \| **Package** \| **Class** \| **Use** \| **Tree** \| **Deprecated** \| **Index** \| **Help** | |  |
| PREV   NEXT | **FRAMES**    **NO FRAMES**     **All Classes** |  |


---
